# Dancers’ Somatic of Musicality

**DOI:** 10.3389/fpsyg.2019.02681

**Published:** 2019-12-05

**Authors:** Niv Marinberg, Vered Aviv

**Affiliations:** Faculty of Dance, The Jerusalem Academy of Music and Dance, Jerusalem, Israel

**Keywords:** dance, rhythm and melody, choreography, movement and music synchronization, somatic response to music

## Abstract

Dancers often perform while synchronizing their movements to music, as required by the choreographer. In this article, we introduce the concept of categorizing choreography (or segments of it), according to its relationship with either the rhythm or the melody of the accompanied music, or with both. We demonstrate this distinction through several examples for each category. In a pilot study, we composed choreographic sequences that were either melodic-based or rhythmic-based and taught them to professional dancers. The results showed that some dancers tend to synchronize their movements better to rhythm and others, to melody. We refer to this tendency as the “dancers’ somatic of musicality.” The findings highlight important differences in the somatic of musicality among dancers, requiring attention from both choreographs and dancers, since these differences have bearing on the way dancers learn, memorize, and perform.

## Introduction

Music and dance are two independent art forms. Nevertheless, most dances are practiced and performed hand-in-hand with music. Listening to music very often triggers a spontaneous bodily response on the part of listeners, irrespective of whether they are dancers or not. This response is expressed as a movement, primarily in tune with the beat or rhythm of the music (Large, 2000; Leman, 2008; Toiviainen et al., 2010). Such somatic response to music has been observed worldwide and in a variety of circumstances, including within the mother and infant relationship, as a factor impacting group cohesion, in tribal dances, and as a personal trait (see, for example, Farnell, 1999; Brown et al., 2005; Luck et al., 2009; Phillips-Silver, 2009; Witek et al., 2014; Honing et al., 2015).

Professional dancers, on the other hand, are expected to move to accompanying music in a specific, choreographed way, rather than spontaneously. Dancers are typically asked by the choreographer to synchronize their movements to certain components of a piece of music, such as its rhythm or melody. This expectation of the choreographer raises questions about the musicality of dancers: is there an inherent difference among dancers’ responses to the various components of a musical piece? Do some dancers synchronize better to rhythm and others to melody? Could such putative differences impact on dancers’ capability to learn or perform certain dance pieces? Could awareness of such differences on the part of the dancer, the choreographer, or the rehearsal manager be used to better adapt the learning process of a dance piece to individual dancers?

It is important to note that contemporary dances may be performed (choreographed) without any accompanying music (see, for example, Waterhouse et al., 2014). Music can also serve as a general background (or noise) to dance; in this case, no specific synchronization of movement to sound is required. However, the aim of this article is to focus, define, and analyze the specific synchronization of choreographed dance to the major components of music: the rhythm and the melody.

In our study, we term the somatic responses of a dancer to various components of music as the “dancers’ somatic of musicality.” Initially, we elaborate on several possibilities of tuning dance sequences to particular components of music, and provide examples of dance sequences choreographed primarily in relation to the melody or to the rhythm of the music. Following this, we examine preliminary data on professional dancers who differ in their somatic responses to various musical components, and offer several practical implications as a result. We note that this study was deliberately conducted in the realistic conditions of a dance studio (rather than in laboratory conditions), because we were interested not only in the theoretical aspects of the dancers’ somatic of musicality but also in the practical implications for dancers, choreographers, and dance rehearsal managers in their daily practice in studios.

## Synchronization of Choreographed Movement Phrases to Different Musical Components

Examining the synchronization between music and dance entails looking at the “onset or continuous action of an event at the same time as another” (Brick and Boker, 2011, p. 285). Thus, to compare the timing between music and dance, one needs to identify key elements (events) in both media. Identification of basic musical components involves detecting the melodic line and the rhythmical pattern (for terminology, see the section “Materials and Methods”). It also necessitates analyzing the musical texture to assess whether or not the melodic line has a distinctive progression throughout the musical piece that is “liberated” from the rhythmical pattern of the sequence. This analysis better determines which of the two facets is prominent in the progression of a particular musical piece.

The identification of key movement components in the choreography is also necessary for analyzing the synchronization between music and dance. People are fundamentally experts in analyzing human motions, including dance (Blake and Shiffrar, 2007; Aviv, 2017). Research has shown that a continuous movement is spontaneously segmented by the observer into movement-related events (Zacks et al., 2007; Kurby and Zacks, 2008). However, researchers have also noted that the segmentation of movement, or phrasing of the movement, differs across individuals (dancers, amateurs, and non-dancers). Segmentation also changes in time with multiple exposure of the same person to the movement phrase (Kahol et al., 2003; Bläsing, 2015). Despite a large variability among viewers with regard to the phrasing of movement, there are common indicators for such phrasing. Among them are the beginnings and endings of movement phrases, positive or negative accelerations, changes in the dynamics of the movement, and changes in the direction of motion (Bläsing, 2015). These common indicators enable the systematical tracking of synchronization between movement and different musical components. Hence, choreography can be defined as either rhythmic-based, melodic-based, or a combination of the two.

### Rhythmic-Based Choreography

Rhythmic-based choreography (RBC) indicates a coordinated relationship between movement and musical rhythm. This choreography is commonplace and easy to specify. In the dance studio, performers frequently use “counts” while synchronizing the dance movement and the rhythm of the music. In RBC, counts serve as a metric tool for segmentation of moments and to regulate their speed. Creating sets of counts serves to coordinate movements with rhythmical patterns. Counting can apply to the movement of different body parts and includes stops between movements. In all cases, the counts must remain consistent with the musical rhythm (Dunn, 2016).

An example of RBC is Mats Ek’s work “Apartment” https://www.youtube.com/watch?v=CjbgKtCYxhQ (12:12–15:00). Another example is Ohad Naharin’s “Trio,” where the music has a clear melodic line and homophonic texture, although the movement phrases do not always adhere to them, adhering primarily rather to the rhythm. https://www.youtube.com/watch?v=D2jmN-A0D4c (27:00–30:10).

### Melodic-Based Choreography

For choreographers who rely on melody when synchronizing dance movements to music, the movement sequence exclusively follows a certain melodic line. In musical pieces with a monophonic texture (see the section “Materials and Methods”), melodic-based choreography (MBC) is more likely, due to the lack of a rhythmic accompanying element. In homophonic and polyphonic textures (see the section “Materials and Methods”), MBC occurs as well. Counting in MBC is not helpful as a tool to facilitate learning the choreography, whereas singing the melody might be. A good example of MBC is Jiri Kylian’s Sarabande, https://vimeo.com/24261649 (11:54–15:29).

### Combined Rhythmic and Melodic-Based Chorography

In rhythmic and melodic-based chorography (RMBC), the movement relates to both the melody and rhythm simultaneously. This type of choreography appears in homophonic as well as polyphonic musical textures. In these textures, the rhythmic patterns are integrated into the melodies, making it inherently impossible to associate the movements with only one of the components (melody or rhythm). In a homophonic texture, the accompanying rhythm is very active and consistent, and the melodic and rhythmic accents are mostly combined and share the same timeline, making them practically inseparable in the dancer’s perception. Alternatively, the choreography may relate to both elements in different time periods (for instance, the legs follow the rhythm while the upper body follows the melodic line). In Matk Ek “Swan lake,” for example, the movement of the solo dancer relates to both components of the music in such a way that it is impossible to differentiate between the melodic or rhythmic patterns: https://www.youtube.com/watch?v=D6rsi1rKBww (4:53–5:40).

## Materials and Methods

Professional dancers are required to demonstrate a high level of precision while simultaneously embodying the clear relationship between movement and music that the choreographer defines. This relationship is not necessarily natural or intuitive to the dancer, who is required to move in a certain way in response to the music by external sources including the choreographer, the rehearsal director, and the teacher.

We explored whether professional dancers have a natural preference to dance to either RBC or MBC, and whether they can switch between types while maintaining the same level of precision. In the pilot experiment described below, professional dancers were asked to perform two short choreographic sequences one intentionally designed as RBC, and the other as MBC. This enabled us to assess the precision of performance in terms of timing, and to analyze preferences.

Participants: Five professional dancers: two females (Amelia and Ofri) and three males (Kelvin, Yotam, and Ohad) aged 18–28 years. All had at least 5 years of formal dance instruction in classical ballet, modern, and/or contemporary dance techniques. All were members of professional contemporary dance companies: the Batsheva Dance Company, the Inbal Dance Company, and the Vertigo Dance Company.

Musical education: The dancers’ level of musical education varied. Amelia had 5 years of piano and 6 years of voice training; Ofri had none. Kelvin had 4 years of piano playing and 1 year of saxophone; Yotam and Ohad had none. All the participants reported having normal vision and were unaware of the purpose of the experiment.

Procedure: All the experiments were conducted in a dance studio in Tel Aviv over a 6-week period, in order to simulate the everyday environment of professional dancers. At the initial meeting, the first author introduced the terms “melody” and “rhythm” to the participants.

Each of the five dancers was asked to freely create one movement phrase of up to 10 s, without any musical reference. The first author then combined the five phrases to create two variations: one version adapted to MBC, and the other to RBC, in relation to specific pieces of music (see below). This procedure was designed to eliminate prior familiarity with a movement genre to avoid biasing the results.

### Experiment 1: MBC

Music source: the Second Movement – Adagietto – of Igor Stravinsky’s (1924)^1^ piano sonata, as it is characterized by mostly a homophonic texture and exemplified by one clear melodic line, with harmonic and rhythmic supportive voices, mostly in the low register (Carr, 2014). The first author choreographed the movements to adhere closely to the melodic lines in the sonata; in other words, the movements illustrated the melodic phrase.

Teaching process: The first author taught the MBC-type phrases, as much as possible avoiding any reference to the rhythmic elements of the music. While teaching, he “sang” the relevant melodies and described the melodic lines as well as the temporal relationship between the movements and the music. After 2 h of rehearsing the material together, the dancers performed the sequence properly and were asked to return after a week to perform individually in front of a camera.

Data collection: A week after studying the 90-s MBC piece, and with no rehearsals in the interim, each dancer rehearsed individually for 30 min without guidelines or corrections, to warm up before performing in front of the camera (ACTIVEON CX Action Camera Camcorder with 5.1 cm LCD). Each dancer performed the MBC twice in succession.

### Experiment 2: RBC

Music source: the same 1924 piano sonata by Stravinsky. However, this experiment was based on the third movement (♩ = 112), which is characterized by a polyphonic texture, mainly between two voices. Each voice leads a different melodic line (Carr, 2014). The sonata’s rhythmic elements are clear, but these change frequently through asymmetric and multiple variations in meter.

Teaching process: the second phrase was synchronized as RBC and focused exclusively on the rhythmic patterns of the sonata. All counts were based on the meter according to the score. The first author taught the RBC piece and practiced it with the dancers using the “clap and count” method. The melodic pattern was not described or emphasized. After 2 h of rehearsing the material together, the dancers were asked to return after a week to perform individually in front of a camera.

Data collection: A week after studying the RBC piece, and with no rehearsals in the interim, each dancer rehearsed individually for 30 min without guidelines or corrections, to warm up before performing in front of the camera (ACTIVEON CX Action Camera Camcorder with 5.1 cm LCD). Each dancer performed the RBC twice in succession.

### Definition of Terms

*Musical Rhythm* is defined as the patterns created by the sequence of notes as music unfolds over time. The length of the note itself, the differences between notes, and the duration of the successive patterns determine the rhythm (Tan et al., 2010). “Rhythm in music is the core element that binds simultaneity and sequentially the sound patterns into structural organizational forms underlying what we consider musical language. In this function, rhythm assumes a critical syntactical role in communicating symbolic, as well as associative, meaning in music” (Thaut, 2013, p. 4).

*Rhythmic Pattern* is defined as a short sequence of events, typically lasting a few seconds and is characterized by the periods between successive onsets of the events. The inter-onset periods are typically simple integer multiples of each other (Timothy and Justus, 2002).

*Melody* is defined by Siu Lan-Tan as the “the experience of a sequence of pitches as belonging together… Melodies are perceived, not in terms of their separate constituent tones, but as coherent units. While each tone of a melody reaches the listeners’ ears as if it were a single bead, listeners ‘thread’ the beads together into continuous strands” (Tan et al., 2010, p. 74). In order to perceive a melodic line the notes need to be identified as a coherent series belonging to each other. Hence, recognizing the existence of a melody involves recognition of an integrated whole (Dowling, 1991; Dowling and Tighe, 2014).

Melody and rhythm are consistently integrated in music, although each of them functions differently as they come together to become music (Peretz and Zatorre, 2005). We discuss this relationship through an analysis of accents and musical textures.

*Accent* increases the perceptual salience of a musical event when that event differs in some way from the surrounding events (Krumhansl, 2004). These events can be melodic or rhythmic, or a combination of the two. Accents put emphasis on a certain facet of the music and consciously or subconsciously influence listeners’ perception of both the rhythm and melody of the music (Tan et al., 2010).

*Musical Texture* refers to the relationship between the melody and harmonics, and the density of the simultaneous layering of different musical components (Kokoras, 2005). In Western music, three main textures have been defined:

– Monophonic texture – music with just one musical voice (Sande, 1993).
– Polyphonic texture – music whose texture is formed by the interweaving of several melodic lines. The lines are independent but together they reverberate harmonically. Each line is (roughly) equally important rhythmically as every other line (Sande, 1993).
– Homophonic texture – usually contains one important leading voice, which is basically the melody, and several secondary accompanying voices supporting the melody harmonically and rhythmically and forming cross-voice associations (Sande, 1993).

Although the perception of texture depends on several factors, including the perception of the rhythmic pattern, texture can last several seconds. In most instances, the texture will remain the same for at least several cycles (Mountain, 1993).

## Results

The 2 h of studying and rehearsing the MBC piece demonstrated that all five dancers clearly understood, and could perform, the movement phrases in a manner that satisfied the first author of this study. With regard to the RBC, all the dancers experienced some initial difficulties in maintaining the sets of counts, probably due to the complexity of the music and choreography. However, after 2 h of rehearsals all dancers performed the choreographic sequence to the satisfaction of the first author.

The main criterion for evaluation of the quality of performance was based on the filmed choreographic pieces; quality was evaluated based on the timing of the movements with respect to the music. Timing refers to the length of motion, stops, and pauses; changes in height level of the dancer; changes of direction in space; changes in the main active body parts; and changes in tempo – all of which were compared to key musical parameters. Timing was examined qualitatively via the “naked” eye of an experienced dance rehearsal instructor (the first author), as is standard practice in professional dance rehearsals. Other movement parameters such as range of motion, effort required for the movement, emotional expressions, or precision of the sequence were not examined because of their marginal relevance to the topic under examination, namely, the evaluation of synchronicity between movement and music.

The results demonstrated that all five dancers could perform the choreography from memory 7 days after first learning it. Individual mistakes occurred mainly at the beginning and ends of movement phrases. The analysis of the filmed performances of MBC and RBC showed variability in synchronization among dancers; this could be classified by referring to two groups. One group (henceforth referred to as the Rhythmic group) was accurate with respect to the rhythmic patterns, whereas inaccuracies were found with respect to the melodic line (Ofri, Kelvin, and Yotam). The reverse was found in the other group (henceforth referred to as the Melodic group), consisting of Amelia and Ohad. Crucially, each dancer exhibited a consistent preference to synchronize the movements either to the rhythm or the melody.

### Video Description

The attached two videos present parts of the documented performances. In each of these videos, two dancers are shown side by side to facilitate a comparison (see the section “Video Description”):

Video 1 https://www.dropbox.com/s/z8g17wldtr8ts8m/MBC4.mov?dl=0

Video 2 https://www.dropbox.com/s/to4ympqouyo5a55/RBC.mov?dl=0

Video 1 shows, as an example, two dancers performing the same MBC. Dancer Amelia (right screen) follows the melodic line precisely, whereas dancer Ofri (left screen) sometimes correlates her movements to rhythmic components in the music. Significant mistakes of Ofri in synchronization of the movement to the melody are marked on the video and on the relevant body part by numbers. Black circles indicate correct timing and red circles indicate incorrect timing.

Video 2 shows the RBC experiment performed by the same two dancers. Amelia (the melodic dancer) on the right screen follows the rhythm only partially. Ofri (the rhythmic dancer) on the left screen follows the rhythm and the counts precisely.

In MBC, when there was a major musical accent and the movement relied solely on the melodic line without any physical accents, some dancers were confused. Mistakes occurred in the case of dancer Ofri each time there was a major rhythmic accent. Rhythmic accents influence the dancer to follow the rhythmic element and not the melody.

Most of the challenges in RBC occur when a clear melodic line does not correlate with the beginning of movement. In this case, moments of mutual accents cannot serve as anchors for the beginning or end of movement. The only way to maintain the relationship between music and movement is to preserve the count system which relies completely on the rhythm. This is similar to our first example of RBC (Mats Ek’s “Apartment”), which requires the dancers to count strictly and refer solely to the rhythmic components. In Video 2, the dancer on the right screen (Amelia) mostly preserved this relationship. However, when the choreography was following the rhythm (i.e., RBC) the dancer unconsciously or by default, synchronized her movements to the melodic line, perhaps considering this line as the primary timing parameter.

In order to statistically validate the grouping of the participants into rhythmic versus melodic dancers, we invited a professional dancer/dance-rehearsal director to serve as a blind coder for this study. The blind coder was not aware of the research question at any time. We then implemented the following procedures: (a) The first author taught the blind coder both dances – the MBC and RBC – using the same method that he had previously used when he taught the dancers. (b) The MBC and RBC were segmented by the first author into movement sequences, according to the movement phrases and their correlation with the music (see the section “Introduction” for more details). The MBC was segmented into five phrases and the RBC was segmented into four phrases (Table 1). (c) Both the first author and the blind coder watched the dance videos (one MBC and one RBC per dancer) and marked the accuracy of the synchronization of movement to music in each of the segments. Mistakes were marked “1” and no mistake “0.” The results are depicted in Table 1. We note that the blind coder was asked to ignore other parameters of the dance performance for evaluation, such as the size of gestures, the precision of directions, and the quality of movement. (d) We compared the evaluation of the two observers using the kappa statistics (Viera and Garrett, 2005). As is clear from Table 1, there is almost perfect agreement between the two observers as indicated by the kappa value of 0.88. Based on this agreement, between two independent dance experts, we may conclude that there is certainly a clear difference in the somatic of musicality among dancers.

**TABLE 1:** Data for calculating the *kappa* score.

**Type of**	**Seq. #1**	**Seq. #2**	**Seq. #3**	**Seq. #4**	**Seq. #5**
**dance = MBC**	**(0–11″)**	**(11–21″)**	**(22–36″)**	**(36–44″)**	**(45–65″)**
**Dancer: Ofri**					
Evaluator: Author #1	1	1	1	1	0
Evaluator: Blind coder	1	1	1	1	0

**Type of**	**Seq. #1**	**Seq. #2**	**Seq. #3**	**Seq. #4**	**Seq. #5**
**dance = MBC**	**(0–11″)**	**(11–21″)**	**(22–36″)**	**(36–44″)**	**(45–65″)**

**Dancer: Amelia**					
Evaluator: Author #1	0	0	0	1	1
Evaluator: Blind coder	0	0	0	1	1

**Type of**	**Seq. #1**	**Seq. #2**	**Seq. #3**	**Seq. #4**	**Seq. #5**
**dance = MBC**	**(0–11″)**	**(11–21″)**	**(22–36″)**	**(36–44″)**	**(45–65″)**

**Dancer: Ohad**					
Evaluator: Author #1	0	0	1	1	1
Evaluator: Blind coder	0	0	1	1	1

**Type of**	**Seq. #1**	**Seq. #2**	**Seq. #3**	**Seq. #4**	**Seq. #5**
**dance = MBC**	**(0–11″)**	**(11–21″)**	**(22–36″)**	**(36–44″)**	**(45–65″)**

**Dancer: Kelvin**					
Evaluator: Author #1	0	1	1	1	0
Evaluator: Blind coder	0	1	1	1	1

**Type of**	**Seq. #1**	**Seq. #2**	**Seq. #3**	**Seq. #4**	
**dance = RBC**	**(0–20″)**	**(20–28″)**	**(29–52″)**	**(52–66″)**	

**Dancer: Ofri**					
Evaluator: Author #1	0	0	0	0	
Evaluator: Blind coder	0	0	0	0	

**Type of**	**Seq. #1**	**Seq. #2**	**Seq. #3**	**Seq. #4**	
**dance = RBC**	**(0–20″)**	**(20–28″)**	**(29–52″)**	**(52–66″)**	

**Dancer: Amelia**					
Evaluator: Author #1	1	1	1	1	
Evaluator: Blind coder	1	1	0	1	

**Type of**	**Seq. #1**	**Seq. #2**	**Seq. #3**	**Seq. #4**	
**dance = RBC**	**(0–20″)**	**(20–28″)**	**(29–52″)**	**(52–66″)**	

**Dancer: Ohad**					
Evaluator: Author #1	0	1	1	1	
Evaluator: Blind coder	0	1	1	1	

**Type of**	**Seq. #1**	**Seq. #2**	**Seq. #3**	**Seq. #4**	
**dance = RBC**	**(0–20″)**	**(20–28″)**	**(29–52″)**	**(52–66″)**	

**Dancer: Kelvin**					
Evaluator: Author #1	1	1	0	0	
Evaluator: Blind coder	1	1	0	0	

**MBC*, melody-based choreography; *RBC*, rhythmic-based choreography. Each choreography piece was divided into movement sequences (Seq. #1, #2, etc.). Score “1” indicates a mistake in synchronizing to music; “0” indicates no mistake. *Kappa* score based on this table is 0.88 (see the text).*

## Discussion

This article introduced the concept of categorizing choreography (or segments of it) according to its relationship to either the rhythm (RBC) or the melody (MBC) of the accompanying music, or to both. It explores differences in the somatic of musicality among dancers, demonstrating that some dancers synchronize their movement better to the rhythm while others do better with the melody.

A pilot experiment was designed to examine whether professional dancers could follow the requirement of a choreographer to synchronize their movement either to the rhythm or the melody of the music. We found a consistent difference among dancers: some dancers (Amelia and Ohad) followed the MBC precisely, whereas the others (Ofri, Kelvin, and Yotam) missed a few timings of the MBC. Conversely, we found that in the RBC, some dancers (Ofri, Kelvin, and Yotam) were temporally accurate with the rhythm, whereas others (Amelia and Ohad) were less precise with the rhythm. This suggests that there are intrinsic differences among dancers’ ability to tune their movement to either the melody or the rhythm of the music. We also gathered participants’ comments about their learning strategies and musicality. To this end, we posed a few questions (see Appendix); the answers of the dancers show a high degree of reflectivity regarding their somatic of musicality. In some cases, their response fits well with our assessment of their personal preference to melody or to rhythm. To quote one (melodic) dancer: “*I always prefer to connect to singing or memorizing rhythms versus using counts. I find counts distracting for the task at hand. When I need to use counts it takes me away from being completely immersed in what I am doing*” (Amelia).

We did not find any correlation between the dancers’ prior musical education and their preferences for either MBC or RBC. It has been claimed that the ability to recognize a melody, as well as a beat in music, is a straightforward characteristic of most individuals (Large, 2000; Honing, 2012; Honing et al., 2015). Therefore, it is likely that all dancers perceived (consciously or unconsciously) both the melody and the rhythm of the music that accompanied their dance. As mentioned in the section “Introduction” (see also Large, 2000), people tend to synchronize their movement to rhythm. Pollatou et al. (2003) found that dancers managed to synchronize their movements to a tambourine rhythm, whereas they were less successful in doing the same when the harmonium guided their movements.

Thus, there is no *a priori* reason to expect a preference on the part some dancers for synchronization of movement to melody; it is therefore surprising to find dancers that do synchronize their movement better to the melody. Studies have suggested that there is a strong reciprocal integration between the auditory and the motor system of the brain during music perception (Zatorre et al., 2007; Phillips-Silver, 2009). However, while there is ample research on the synchronization of movement with beat and rhythm, there is scant research on the synchronization of movement with melody, let alone how this occurs during dance (Brown et al., 2005; Phillips-Silver, 2009). We note that some preliminary support for the separation of brain activities during rhythm-related movement from brain activities during non-rhythm-related movement was reported (Brown et al., 2005; Phillips-Silver, 2009). Some experiments demonstrated that the perception of rhythm may influence the perception of melody (Prince, 2014). The consistency of the somatic of musicality of dancers, as well as the reasons for it, needs further investigation.

### The Notion of Dancer’s Somatic of Musicality in Light of Current Entrainment Theories

Entrainment refers to the temporal attunement of two (or more) independent organisms resulting in coordinated rhythmic behavior (Large, 2000; Phillips-Silver et al., 2010; Clayton, 2012). There are several types of entrainment including the coordinated movements between people (Knoblich and Sebanz, 2008; Schmidt et al., 2011; Phillips-Silver and Keller, 2012; Chauvigné et al., 2014; Waterhouse et al., 2014); entrainment between people and an external signal such as entrainment to circadian rhythm (chronobiology) or to a metronome; and entrainment between movement of people and music (e.g., Chauvigné et al., 2014). All these types of entrainment are frequently found in dance which, by its nature as a performing art based on human movement, initiates situations for a variety of types of synchronizations.

The present study focuses on a particular subcase of entrainment conditions, whereby the movement is (i) choreographed [non-spontaneous; for spontaneous movements, see Burger et al. (2014), and the section “Introduction”]; (ii) is synchronized to a particular component of the music (rhythm or melody); and (iii) is performed by a solo dancer (versus a group performance). The latter implies that there could not be entrainment to one or more additional dancers. Our study shows that under the three conditions denoted above, professional dancers vary in their tendency to synchronize to music. Some dancers tend to be better entrained to the music’s rhythm, and others to its melody. We thus uncovered a refined resolution of movement to music entrainment skills.

It is worth stressing that this pilot experiment measured the execution of synchronization as embedded in a dancer’s long-term memory. Research has indicated that both learning and performance of dance sequences are enhanced when taught in the presence of music (Starkes et al., 1987; Stevens et al., 2011; see also Appendix). It has also been argued that music functions as contextual features, mental “landmarks,” that facilitate forming associations between the music and movement sequences. The presence of the same music used while learning the movement sequence has been shown to cue the recall of the timing and dynamics of movements during execution (Starkes et al., 1987; Stevens et al., 2009, 2011; Bläsing et al., 2012). It remains unclear whether tagging the movement to the melodic line or to the rhythm of the music was part of the long-term memory process of the dance sequence, or whether the motor execution of the dance was linked to the dancers’ tendency to respond more to melody or rhythm.

We noticed that immediately after learning, all dancers performed both the RBC and MBC sequences accurately, whereas a week later, there were noticeable differences between the rhythmic and melodic dancers. These findings could be interpreted in light of the current theories on dance and entrainment (see the section above and Waterhouse et al., 2014). Indeed, it was suggested that interpersonal entrainment has a strong effect on the synchronization of movement between dancers during dance learning and performing. Alternatively, these results could be interpreted in light of the powerful effect of imitation as a tool both for learning the dance and for performing it (Brass and Heyes, 2005; Brown and Parsons, 2008; Leisman and Aviv, 2020). Additional research is required in order to uncover the mechanisms that lead to the evident differences between the group and the solo performances.

The terminologies that we introduced – those of the somatic of musicality and the dancers’ preference for MBC or RBC – have important implications. Professional training and educational systems for dancers can enhance their development through the use of better articulated distinctions and categorizations of the music-based choreography. Classifying and being aware of dancers’ somatic preferences would be beneficial for practical learning and for coping with the limitations of dancers and the differences between them. Being cognizant of the variability of dancers’ somatic of musicality can enable the choreographer, dancer, dance rehearsal instructor, and teacher to work together to enhance unification, leading to greater precision in dance performance.

## Data Availability Statement

The datasets generated for this study are available on request to the corresponding author.

## Ethics Statement

The studies involving human participants were reviewed and approved by the Institutional Ethics Committee of the Jerusalem Academy of Music and Dance, number 0327. The patients/participants provided their written informed consent to participate in this study. Written informed consent was obtained from the individual(s) for the publication of any potentially identifiable images or data included in this article.

## Author Contributions

NM and VA conceived and designed the experiments and analyzed the results. VA wrote the manuscript. NM performed the experiments. This work was part of Nm’s M.A. project under the supervision of VA.

## Conflict of Interest

The authors declare that the research was conducted in the absence of any commercial or financial relationships that could be construed as a potential conflict of interest.

## Footnotes

1 https://en.wikipedia.org/wiki/Piano_Sonata_(Stravinsky)

## Appendix

### Dancers’ Comments About Learning Strategies and Musicality

We questioned the dancers participating in this study, asking them for freestyle answers. Below are the questions and the respective answers:

Q. Do you prefer, in general, to synchronize the choreography by counts or by “singing,”/memorizing the music? Is your answer consistent, or does it depend on the type of choreography? To what degree is it typical for you?

Ohad: I think it depends on the music for me, but generally I prefer singing or memorizing the music. Often, I learn the counts, but then at some point, I start remembering the music they sit on and I stop counting. Sometimes, with a more complex musicality, I have to count even music that I know; otherwise, I don’t manage. I think that eventually I am more inclined to rely on memorizing the music, but I also work with counts and I also rely on them, especially when I learn new choreography.

Amelia: I always prefer to connect to singing or memorizing rhythms versus using counts. I find counts distracting for the task at hand. When I need to use counts, it takes me away from being completely immersed in what I am doing.

Ofri: For me, it definitely depends on the type of music and the type of choreography, as to whether I prefer counts or “singing.” I would say that if the music is countable in the classic eight counts of dancers, and the choreography relates clearly to these bars, then it would be hard for me not to count. With music that is more complex, I would naturally turn to singing and memorizing it. Also, I would say that it is easy for me to get the counts if I am required to, but if I am not asked to do that I would not choose to work with them of my own volition.

Kelvin: I try to understand the musicality of choreography through a variety of tools, including counts, singing, and memorizing the music. It depends on the choreography and the music – some music is more complicated to count and easier to sing, and vice versa. Often, to understand musical structure and phrasing, I will try to count through the music without the choreography, to find anchors and landmarks within the music that are easy for me to recognize or catch if I get lost. And then I will count some parts of the choreography and use the anchors for other parts. My aim is to know the music and choreography thoroughly enough to be able to sing and count my way through it. I’ve found that when I feel insecure or stressed, I tend to rely on counts, though this sometimes prevents me from really “listening” to the music and its layers.

Yotam: Regarding the first question, I believe that, for me, there is no consistent answer. Usually at first, I try to learn the choreography by counting, but through the process, I have noticed I tend to mix the two, counting and singing/memorizing the music. I feel that it really varies with each choreography and piece of music. Sometimes, I can really feel the rhythm of the movements and sometimes I really depend on moments in the music.

Q: Do you find it difficult to understand the method some choreographers/rehearsal directors apply to teach the movement? Please explain why.

Ofri: Usually, it is not hard for me to understand methods of choreographers or rehearsal directors concerning teaching movement or musicality. Sometimes there could be a gap between my first understanding about movement, or my preferences about it, and the way the person in front of me decides to teach it. In those cases, I am mostly able to compensate over this gap, to “translate” the way I think about the movement to the way the person teaching it does. Or, in difficult rare cases, I copy until I “make” it.

Kelvin: I’ve found that if a choreographer or rehearsal director is clear, specific, and creative enough, I can usually follow along with little trouble. That is asking a lot, though, and sometimes I do wonder if a specific choice of teaching is the most efficient or effective. But in times of confusion, I try to translate and navigate my own way through the material.

The methods that I have the most trouble with are the dry instructions/counts with no sense of qualities or colors, or digging too much into the material so that all of the associations or connotations of the movement become stronger than the source. There’s a nice window between these two places; that’s what I find the easiest to learn and understand.

Yotam: From my past experience with learning movements from many different people I do find it difficult sometimes. I have noticed that for me it is easier to understand movements if I’m learning them in different layers such as texture, touch, musically, etc. It is better for me to slowly get into movement material than to learn everything fast and as one chunk.

Q: Did you find any difference between studying the two pieces in the study? Which of the two was more natural or clear to you? (If you remember)

Ofri: My memory about studying the phrases is a bit vague. I would say that both phrases were challenging because neither was very classic synchronization of movement to music. Classic in the sense that I usually don’t come across this kind of work. In the phrase when we were singing, I remember both enjoying some great moments of synchronizing that were very clear, and some moments I wasn’t sure about, when I almost felt as if it was hard for me to anticipate the music. In the phrase when we were counting, I remember feeling like it was solving a puzzle, like it was a difficult game of matching the movements correctly on the counts. I enjoyed this game. But I would say it was more brainy than “dancy.”

Kelvin: Hard for me to remember … but I’ll try. I found that both were equally natural or unnatural to me. I don’t consider myself a quick or intuitively musical dancer, and it can take me a while to really understand music. I began to understand that what was most effective for me was combining the different methods or frames for musical learning so that I could understand the music better, and also how the choreography was in conversation with the musical structure. When I understood or made up some underlying logic or system, it was much easier to execute and feel secure in that execution.

Yotam: I don’t remember clearly the two pieces but here I can guess and say that it was probably easier and more natural for me to learn the Nina Simone piece. Definitely because of the words of the song which I could hold on to, and also because it was more repetitive than the other piece by Stravinsky. The Nina Simone song piece was much easier to sing and memorize compared to the Stravinsky piece.
